# Home Telehealth Uptake and Continued Use Among Heart Failure and Chronic Obstructive Pulmonary Disease Patients: a Systematic Review

**DOI:** 10.1007/s12160-014-9607-x

**Published:** 2014-04-25

**Authors:** Sarah L. Gorst, Christopher J. Armitage, Simon Brownsell, Mark S. Hawley

**Affiliations:** 1 Department of Psychology, University of Sheffield, Western Bank, Sheffield, S10 2TP UK; 2 Manchester Centre for Health Psychology, School of Psychological Sciences, Manchester Academic Health Sciences Centre, University of Manchester, Coupland Street, Oxford Road, Manchester, M13 9PL UK; 3 The James Cook University Hospital, Marton Road, Middlesbrough, TS4 3BW UK; 4 Centre for Assistive Technology and Connected Healthcare (CATCH) and School of Health and Related Research (ScHARR), University of Sheffield, Regent Court, Regent Street, Sheffield, S1 4DA UK

**Keywords:** Telehealth, HF, COPD, Systematic review, Uptake

## Abstract

**Background:**

Home telehealth has the potential to benefit heart failure (HF) and chronic obstructive pulmonary disease (COPD) patients, however large-scale deployment is yet to be achieved.

**Purpose:**

The aim of this review was to assess levels of uptake of home telehealth by patients with HF and COPD and the factors that determine whether patients do or do not accept and continue to use telehealth.

**Methods:**

This research performs a narrative synthesis of the results from included studies.

**Results:**

Thirty-seven studies met the inclusion criteria. Studies that reported rates of refusal and/or withdrawal found that almost one third of patients who were offered telehealth refused and one fifth of participants who did accept later abandoned telehealth. Seven barriers to, and nine facilitators of, home telehealth use were identified.

**Conclusions:**

Research reports need to provide more details regarding telehealth refusal and abandonment, in order to understand the reasons why patients decide not to use telehealth.

**Electronic supplementary material:**

The online version of this article (doi:10.1007/s12160-014-9607-x) contains supplementary material, which is available to authorized users.

## Background

Heart failure (HF) and chronic obstructive pulmonary disease (COPD) place considerable burden on patients and healthcare systems through repeated emergency department visits and lengthy hospital admissions [1, 2]. The incidence of HF and COPD is increasing [3–5], and the associated costs are substantial. In 2012, the combined total annual direct cost of HF and COPD in the USA was estimated to be over $50 billion [6, 7]. Furthermore, HF and COPD patients report significant impairments in their physical and social functioning [8–11]. Quality of life among patients with HF and COPD is significantly lower than that of the general population [12–14] and that of patients with other chronic diseases [9, 15, 16]. As the population ages and the incidence of HF and COPD escalates, it has been suggested that telehealth may become the only cost-effective means of maintaining and enhancing the quality of care [17–19].

Telehealth is a comprehensive concept that encompasses the transfer and exchange of health information through electronic devices. The focus of the present review is home telehealth, which involves the remote delivery of health-related services via information and communication technologies between a patient and healthcare professionals, to assist in the monitoring and management of a patient’s health condition [20, 21]. The review will not focus on mobile phone-based telehealth interventions, because at present mobile-based systems are not a mainstream means of delivering telehealth to HF and COPD patients. Compared with traditional approaches, telehealth may deliver enhanced care to HF and COPD patients by providing early warning of health status deterioration, thereby avoiding negative health outcomes [19, 22–24]. Telehealth may also reduce costs by decreasing rehospitalization rates [25, 26]. Previous systematic reviews have indicated that compared with usual care, telehealth shows benefits in terms of reduced emergency department visits, hospital admissions, and mortality rates, enhanced quality of life, and improvements in patient knowledge and self-care [27–29], although some recent large studies have not shown such benefit [21, 30, 31].

Despite mixed evidence of benefit, there is a strong policy push to introduce telehealth in the UK and Europe. The UK Department of Health believes that telehealth and telecare (services that enable people to live independently and securely in their own homes) could benefit the lives of at least three million people with long-term conditions and/or social care needs. The rationale is that, if implemented effectively as part of a whole system redesign of care, then telehealth and telecare can ease pressure on long-term UK National Health Service costs and improve people’s quality of life through enhanced self-care in the home environment [32]. Large-scale deployment of telehealth in the UK or in Europe is, however, yet to be achieved.

Patient acceptance has been identified as one of the most important influences on the future implementation of telehealth [33], and there is a perception that many patients refuse or quickly abandon telehealth [34, 35]. Despite this, the proportion of patients who refuse or abandon telehealth is largely unknown. Research is needed to quantify the rates of patient uptake, refusal, and abandonment of telehealth, to understand the number of patients who are willing to accept and use it. Research also needs to explore patient beliefs and perceptions about telehealth to try and explain why patients decide to take up, refuse, abandon, or sustain their use of telehealth.

A systematic review was undertaken to consolidate current knowledge on: (a) the rates of uptake, refusal, and abandonment of home telehealth by patients with HF or COPD, and (b) the factors that influence whether patients with HF or COPD do or do not accept and use telehealth.

## Methods

The review was conducted in accordance with the Cochrane guidelines for systematic reviews [36].

### Search Strategy

The following databases were searched for relevant studies published without date limits up to the date of the search (September 2013): the Cochrane CENTRAL Register of Controlled Trials, Cumulative Index to Nursing and Allied Health Literature, MEDLINE, PsycInfo, and the ISI Web of Knowledge/Science/Conference Proceedings Citation Index. To be as comprehensive as possible, it was necessary to include a wide range of free-text terms for each of the concepts. The following population search terms were used: “Heart Failure” OR “HF” OR “Chronic Obstructive Pulmonary Disease” OR “COPD.” The following intervention search terms were used: “telehealth” OR “telemedicine” OR “telecare” OR “telehomecare” OR “telemonitoring” OR “telemanagement” OR “teleconsultation” OR “telecommunications” OR “remote consultation” OR “remote monitoring” OR “assistive technology” OR “ehealth” OR “telenursing.” The following outcome terms were used: “uptake” OR “adoption” OR “refusal” OR “abandon*” OR “accept*” OR “embrace” OR “reject*” OR “decline” OR “beliefs” OR “perceptions” OR “facilitators” OR “barriers” OR “obstacles” OR “challenges” OR “sustained use” OR “maintenance.” Hand-searching reference lists of other systematic review articles was also conducted [21, 27–29], as research has found this method to be valuable in identifying studies for inclusion in systematic reviews of healthcare [37]. General Web searching was not conducted, as there is little empirical evidence as to the value of using general Internet search engines to identify potential studies [38]. All lists of citation results were generated and exported into MENDELEY (Mendeley, Ltd. London, UK, www.mendeley.com).

### Inclusion and Exclusion Criteria

A study was eligible for inclusion if it described an intervention that specifically utilized technology as a means of delivering healthcare to patients with a diagnosis of HF and/or COPD in their own homes or in a residential care home. Telehealth had to be a core component of the intervention. Interventions used by the patient with no interaction or input from a health professional or facilitator were excluded. Technology using mobile phone-based interventions was also excluded, as the focus of the review was home telehealth. Studies were included in the review if they mentioned any details relating to patient acceptance, abandonment, or perceptions of telehealth. Randomized controlled trials, correlational survey research, and observational research were eligible for inclusion. Published conference proceedings were included where sufficient data were provided on population, intervention, and outcomes. Nonprimary research and dissertations were excluded. HF and/or COPD patients confirmed by medical records or by medical practitioner were eligible for inclusion. Hospitalized patients and those with acute exacerbations of symptoms were excluded. Participants had to be aged 18 years or above. Data to be extracted for HF and/or COPD patients had to be presented in isolation from patients with a different diagnosis (see Table 1).

**Table 1:** Study inclusion and exclusion criteria

Inclusion criteria	Exclusion criteria
Type of studies	
Randomized controlled trials, correlational survey research and observational research	Commentaries, editorials and expert opinion, literature and systematic reviews, letters, and other nonprimary research
Published conference proceedings, where sufficient data was provided on population, intervention, and outcomes	Dissertations and papers written in non-English language
Types of participants	
HF and/or COPD patients confirmed by medical records or by medical practitioner	Hospitalized patients and those with acute exacerbations of symptoms
Living at home or in a residential care home	
Aged 18 years or above	
Data to be extracted presented for HF and/or COPD patients in isolation from data from patients with a different diagnosis	
Types of interventions	
Utilized technology as a means of delivering healthcare to patients with a diagnosis of HF and/or COPD in their own homes	Interventions with no interaction or input from a health professional or facilitator
Technology had to be electronic and use either POTS (plain old telephone service) or broadband connection	Interventions using telecommunication technologies primarily for educational or administrative purposes and not linked to direct patient care
Health or care involved healthcare delivery, education, advice that involved a healthcare provider/professional within data transfer	Technology using mobile phone-based interventions
Telehealth had to be a core component of the intervention	Interactions at GP practices or hospitals/clinical settings, residential homes, prisons, or other institutions
Points of healthcare delivery were limited to: home, sheltered housing, extra care, and nursing home	
Types of outcome measures	
Any details relating to patients acceptance, abandonment, or perceptions of telehealth	

*HF* heart failure, *COPD* chronic obstructive pulmonary disease, *GP* general practitioner

### Review Procedure

Two members of the study team (SLG and CJA) independently screened the titles and abstracts. Full papers were obtained if either reviewer did not exclude the paper based on the abstract or title. Full texts of papers were obtained and read by one author (SLG). The relevance of each study was assessed according to the predefined inclusion criteria.

### Data Extraction

Data from the included studies were extracted by author SLG into a data extraction sheet prepared for this study (see Electronic Supplementary Material (ESM) 1) and checked for accuracy by author SB.

### Data Analysis

A narrative synthesis of the data, primarily in terms of study design, population, type of intervention, rates of uptake, refusal, and abandonment and barriers to, and facilitators of, acceptance and sustained use of telehealth, was carried out (ESM 2). Thematic analysis was used to identify the most important and recurrent themes relating to the barriers and facilitators across the multiple studies. The analysis was developed in an inductive manner, without a set of a priori themes to guide data extraction and analysis.

### Thematic Analysis

Themes were separated into two categories: Those aspects of the intervention that were judged to act as barriers to telehealth and those aspects of the intervention that were judged to act as facilitators of telehealth. Barriers were defined as any negative factors that may lead to patients declining or abandoning telehealth. Facilitators were defined as any positive factors that may lead to patients deciding to take up or sustain using telehealth. There needed to be multiple (≥2) occurrences of positive or negative details within and between papers, in order for them to be considered a barrier or facilitator. To gain insight into patient perceptions of the barriers to and facilitators of telehealth acceptance and adherence, the themes were listed in two tables, linking the barriers and facilitators with the studies in which they were reported. From these tables, it was possible to see which barriers and facilitators were reported most frequently. Rather than categorizing themes separately for HF and COPD patients, they were combined into a single category, as several previous studies have combined the results for HF and COPD patients regarding the impact and perceptions of telehealth [19, 22, 39]. “Ideas Webbing” was used to conceptualize and explore connections among the themes. This approach uses figures/diagrams to develop a visual picture of possible relationships across study results [40].

### Assessment of Methodological Quality

Methodological quality of studies was not used as an inclusion criterion for the review, due to the diversity in study design [41] and because the focus of the review was on patient perceptions of the intervention as opposed to the effectiveness of the intervention per se. Excluding studies based on quality would have limited understanding of this objective. This thematic analysis consequently reflects a purposeful decision to deviate from focusing on methodological quality as a criterion for inclusion. Nevertheless, giving equal weighting to studies of good rigor and those with methodological flaws could lead to drawing out inappropriate conclusions, so the quality of papers and their impact has been commented upon in accordance with the Critical Appraisal Skills Program critical appraisal checklists, which covered rigor, credibility, and relevance. Studies were rated independently by one author (SLG) and checked by a second author (SB). A total methodological quality score was calculated for all papers. Studies scoring in the 75th percentile or higher on quality were categorized as “high-quality” studies. Studies scoring between 50 and 75 % were rated as “moderate quality.” Studies scoring lower than 50 % were considered “low quality” [42].

## Results

### Study Selection

**Fig. 1:**
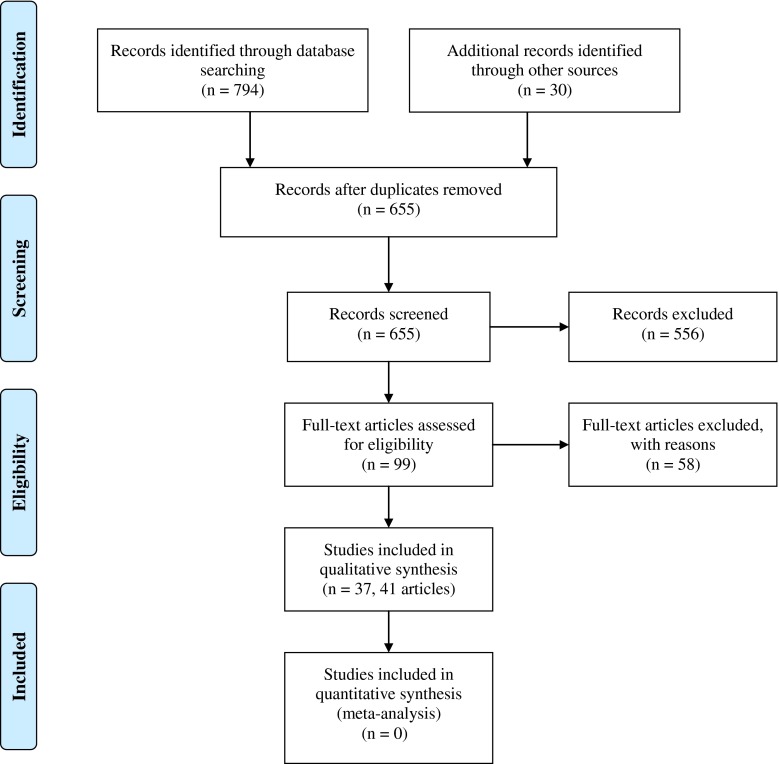
PRISMA 2009 flow diagram

The initial cross-database search yielded 824 articles (Fig. 1). In total, 169 articles were immediately excluded, for reasons including duplication and type of article (e.g., dissertations, editorials, and systematic reviews). The titles and abstracts of 655 articles were screened (by SLG and CJA) and 556 were excluded, as they did not meet the inclusion criteria. Full texts were obtained for 99 articles, of which 58 were excluded (ESM 3). The most common reasons for exclusion at this stage were: data for HF and/or COPD patients were not presented in isolation; no details on acceptance or abandonment; or lack of detail on patient perceptions. This resulted in a total of 41 articles [19, 22–24, 43–79] describing 37 individual studies, being eligible for inclusion in the review (ESM 4).

### Characteristics of Selected Studies

Of the 37 studies that met the inclusion criteria, 13 were randomized controlled trials (RCT: [43, 45, 46, 49, 58, 60–63, 67, 69, 73, 74, 78]), nine employed mixed methods [19, 24, 47, 52, 53, 55, 56, 64, 70, 71, 77], eight were qualitative [22, 23, 44, 54, 57, 59, 66, 76], and seven were cohort studies [48, 50, 51, 65, 68, 72, 75, 79] (see ESM 5 for definitions of study designs).

### Characteristics of Study Populations

Of the 37 studies, 22 included patients with HF only, 11 included patients with COPD only, and 4 studies included patients with both HF and COPD. The number of study participants ranged from 4 to 420 with an overall mean of 78 participants and a median of 50 (37 studies). The number of participants provided with telehealth, or whose perceptions of telehealth were assessed, ranged from 4 to 195 with an overall mean of 51 participants and a median of 30 (37 studies) (ESM 4). Descriptions of study populations were not always comprehensive. Mean percentages have been calculated and presented from studies with the available data. The mean age of all participants was 65 years (30 studies: [19, 22–24, 43–47, 49–51, 54–57, 61–63, 65–69, 72–76, 78, 79]); the mean age for participants offered telehealth was 68 years (28 studies: [19, 22–24, 43–47, 50, 51, 54–57, 61, 62, 65–69, 72–76, 78, 79]). Thirty-one studies reported the gender of the total sample (mean 62 % men and 38 % women: [19, 22, 24, 43–48, 50, 51, 54–57, 60–63, 65–69, 71–76, 78, 79]); thirty studies recorded an average of 59 % men (41 % women) offered telehealth [19, 22, 24, 43–48, 50, 51, 54–57, 60–62, 65–69, 71–76, 78, 79]. Regarding ethnicity, 56 % of the total samples were white and 58 % of people offered telehealth were white (11 studies: [22, 24, 45, 48, 55, 61, 66–68, 73, 74]). In terms of living situation, 66 % of the total sample were residing with their spouse, partner, relative, or caregiver and 34 % were living alone (12 studies: [19, 22, 24, 45, 47–49, 57, 62, 67, 71, 78]). Similarly, 64 % of people offered telehealth were residing with their spouse, partner, relative, or caregiver and 36 % were living alone (11 studies: [19, 22, 24, 45, 47, 48, 57, 62, 67, 71, 78]). Ten studies provided education details for participants, and found the majority of both the total sample and those offered telehealth to be high school educated or above (62 and 67 %, respectively: [22, 45, 48, 55, 60, 65–67, 73, 78]).

Out of the 22 studies that included patients with HF only and the 4 studies that included patients with both HF and COPD, 16 studies reported details about the health status of participants. Thirteen studies reported details about the average stage of HF for all participants, ranging from mild (four studies: [49, 51, 65, 74]) to moderate (nine studies: [22, 45, 47, 48, 55, 66, 68, 69, 73]). Twelve studies reported details about the average stage of HF for telehealth participants only: the reported stages were mild (two studies: [51, 65]), moderate (nine studies: [22, 47, 48, 55, 66, 68, 69, 73, 74]), and severe (one study: [45]). Out of the 11 studies that included patients with COPD only and the 4 studies that included patients with both HF and COPD, 9 studies reported details about the average COPD severity grade of all participants and telehealth participants only. The reported grades were: mild (one study: [44]), moderate (one study: [57]), and severe (seven studies: [43, 46, 52, 53, 62, 67, 75, 76]). Twelve of the 37 included studies reported no details about the health status of participants [19, 23, 24, 54, 56, 59, 60, 64, 71, 72, 77, 78].

### Intervention Characteristics

All of the 37 studies included in the review featured either an intervention, a demonstration of a telehealth device, or patient interviews discussing perceptions of a telehealth intervention. The interventions included: Remote monitoring and transmission of physiological data, assessment of health symptoms, nurse telephone contact or video consultation, disease specific education, health questions, and reinforcement of self-management behaviors (ESM 4).

### Participant Recruitment

Recruitment context varied across the included studies. Participants were recruited from hospitals and/or medical centers (14 studies: [19, 23, 43–46, 50, 51, 58, 60, 61, 69, 72, 74, 75, 78]), healthcare systems or databases (4 studies: [49, 52, 53, 62, 68, 70]), HF units/clinics (3 studies: [24, 65, 79]), and a Medicare Coordinated Care Demonstration project (1 study: [66]). Eight studies recruited patients who were already using or had previously used some form of telehealth service [22, 47, 48, 54, 57, 59, 71, 76]. Another study recruited participants through recruitment announcements that were sent to email distribution lists and online support groups for COPD patients [67]. The recruitment context for six of the studies was not stated [55, 56, 63, 64, 73, 77].

### Participant Refusals

Eight studies reported participant refusal rates for those offered telehealth only, (see Table 2). On average, the studies reported that 32 % (range, 4–71 %) of participants refused telehealth [45, 48, 50, 51, 61, 63, 65, 68, 72]. Five out of the eight studies provided reasons for refusal, with the most common reasons being: Participants not interested and/or believing monitoring to be unnecessary [45, 50, 51, 61, 65, 72]. Three studies reported some demographic information for refusers: Mean age ranged from 68 to 70 years, females accounted for 31 to 53 %, 88 % of refusers were white, 45 % were married, and 80 % were classified in New York Heart Association functional class II [50, 51, 61, 72].

**Table 2:** Details on participant refusals and withdrawals from telehealth interventions

Study	Refusals (R)	Reasons	Withdrawals (W)	Reasons	Demographics
1. Antoniades et al. [43]	Occurred before randomization	N/A	4/22 (18 %)	Placement; moved interstate; cancer; comorbidities	Not reported
2. Bedra et al. [44]	N/A	N/A	N/A	N/A	N/A
3. Bowles et al. [45]	24/101 (24 %)	Refused equipment upon arrival—“too sick to bother;” concern over nurses altering phone systems to connect equipment; and discouraged from participating by nurses, as nurse had to set up equipment	7/77 (9 %)	Not reported	Not reported
4. Casas et al. [46]; Garcia-Aymerich et al. [58]	Occurred before randomization	N/A	5/65 (8 %)	Palliative care (3); change of address (2)	Not reported
5. Clark et al. [47]	Not reported	N/A	19/79 (24 %)	Poor health; difficulty understanding English; transfer to nursing home; found program unacceptable	Not reported
6. Delaney and Apostolidis [48]	11/55 (20 %)	Not reported	Not reported	N/A	Not reported
7. de Lusignan et al. [49]	Occurred before randomization	N/A	1/10 (10 %)	Found monitoring and video consulting “overwhelming”	Not reported
8. Domingo et al. [50, 51]	70/211 (33 %)	Not interested (35); does not feel capable (15); depression (5); long periods away from home (3); other (4); and retracted consent before installation of the telemedicine system (8)	22/97 (23 %)	Patient rejection of use of the system (13); incidents related to the telemonitoring equipment, including: lack of internet coverage (5); inability to complete the requirements of telemonitoring (3); and severe functional deterioration	R: mean age = 68.9; sex = 30.7 % female; New York Heart Association class II = 80 %, III = 18.6 %, IV = 1.4 %); W: not reported
9. Fairbrother et al. [52, 53]; Pinnock et al. [70]	Not reported	N/A	Not reported	N/A	N/A
10. Fairbrother et al. [54]	N/A	N/A	N/A	N/A	N/A
11. Finkelstein et al. [55]	N/A	N/A	N/A	N/A	N/A
12. Finkelstein and Wood [56]	N/A	N/A	N/A	N/A	N/A
13. Gale and Sultan [57]	Not reported	N/A	Not reported	N/A	N/A
14. Johnston and Weatherburn [59]	N/A	N/A	N/A	N/A	N/A
15. Kim et al. [60]	Not reported	N/A	63/207 (30 %)	Withdrawing consent; exacerbation of disease; inability to manage monitoring devices; terminating communication	Not reported
16. Kulshreshtha et al. [61]	40/82 (49 %)	Too busy; unsure of the technology; worried that monitoring would make them feel disabled; physician dislike of technology; fear of information overload; physician doubt that patient would cooperate	4/42 (10 %)	Moved to another city (2); stopped sending readings (2)	R: Mean age = 67.9; Sex = 45 % male; Race = 87.5 % white W: Not reported
17. LaFramboise et al. [22]	Not reported	N/A	48 %	57 % (of 48 % withdrawals) did not want to use health buddy	Not reported
18. Lewis et al. [62]	Occurred before randomization	N/A	1/20 (5 %)	Too cumbersome, when patient wanted to travel	Not reported
19. Louis et al. [63]	7/169 (4 %)	Not reported	8/162 (5 %)	Asked for equipment to be removed (5); discontinued recording (3)	Not reported
20. Lovell et al. [64]	Not reported	N/A	Not reported	N/A	N/A
21. Maric et al. [65]	63/89 (71 %)	Not interested (26); could not come for follow-up (10); “too busy ” (9); refused for other reasons (11); could not be contacted or chose not to enroll for nonspecified reasons (7)	3/20 (15 %)	Could not get used to Website; schedule change; unknown	R: not reported; W: age = 54; sex = 100 % male; marital status = 67 %; New York Heart Association class I = 33 %, II = 67 %
22. Nahm et al. [66]	N/A	N/A	N/A	N/A	N/A
23. Nguyen et al. [67]	Occurred before randomization	N/A	8/26 (31 %)	Unable to access Website (4); schedule conflict; recurrent angina; moved from area; lost interest	Not reported
24. Piette et al. [68]	57/173 (33 %)	Not reported	Not reported	N/A	Not reported
25. Pinna et al. [69]	Not reported	N/A	18/195 (9 %)	Not reported	Not reported
26. Radhakrishnan et al. [71]	N/A	N/A	N/A	N/A	N/A
27. Rahimpour et al. [23]	N/A	N/A	N/A	N/A	N/A
28. Schmidt et al. [72]	30/62 (48 %)	Believed there was no need for medication compliance monitoring (30)	3/32 (9 %)	Not reported	R: age = 70.12; sex = 53 % female; marital status = 44.8 % married; W: not reported
29. Seibert et al. [73]	Not reported	N/A	3/13 (23 %)	Not reported	Not reported
30. Spaeder et al. [74]	Not reported	N/A	1/25 (4 %)	Not reported	Not reported
31. Trappenburg et al. [75]	Not reported	N/A	26/101 (26 %)	Technical problems (11); lack of motivation to participate (10); moving out of area and unable to continue telemonitoring (5)	Not reported
32. Ure et al. [76]	Not reported	N/A	Not reported	N/A	N/A
33. Venter et al. [77]	Not reported	N/A	Not reported	N/A	N/A
34. Whitten and Mickus [19]	Not reported	N/A	46/83 (55 %)	Data issues; agency discontinuity; unwillingness to comply with pre- and postdata collection	Not reported
35. Whitten et al. [24]	Not reported	N/A	Not reported	N/A	N/A
36. Wong et al. [78]	Occurred before randomization	N/A	2/30 (7 %)	Not reported	Not reported
37. Wu et al. [79]	Not reported	N/A	32/58 (55 %)	Not reported	Not reported

### Participant Withdrawals

Twenty-one studies reported participant withdrawals from the study, relating to people offered telehealth only (see Table 2). On average, the studies reported that 20 % (range, 4–55 %) of participants who agreed to participate subsequently withdrew [19, 22, 43, 45–47, 49–51, 58, 60–63, 65, 67, 69, 72–75, 78, 79]. Fourteen out of the 21 studies provided reasons for withdrawal, with the most common reasons being: Participants not wanting to use the telehealth device; health deterioration; and technical problems [19, 22, 43, 46, 47, 49–51, 58, 60–63, 65, 67, 75]. One of the 21 studies reported details about demographics and found people who withdrew had a mean age of 54 years, 100 % were male, 67 % were married, and 67 % were classified in New York Heart Association functional class II [65].

### Patient Barriers to Telehealth

Patient barriers to telehealth were reported in 17 studies (ESM 6: [19, 22–24, 44, 47, 49, 54, 56, 59, 67, 69, 71–73, 76, 79]). Seven individual barriers were recorded: Technical problems, believing telehealth to be unnecessary, preference for in-person care, technology anxiety, difficulty remembering to interact with system, need for technical support, and finding telehealth to be a repetitive process (see Table 3).

**Table 3:** Barriers to telehealth

Barrier	Definition	Paper number
Technology related	Barriers relating to technology, which prevent patients from using or makes it difficult to use telehealth	
Technical problems	Issues relating to equipment and technology, including: difficulty connecting to system, equipment failure, loss of data, usability challenges, and failed transmissions	2, 5, 7, 10, 12, 14, 23, 25, 26, 32, 34, and 37
Technology anxiety	Tendency to feel hesitant, nervous or uneasy about using the technological equipment	26, 27, and 34
Technical support	Requiring assistance, to use or continue using technical equipment	23 and 27
Telehealth process	Barriers relating to the process of telehealth, which prevent patients from using or makes it difficult to use telehealth	
Believing telehealth to be unnecessary	Not understanding the purpose of telehealth and considering telehealth monitoring to be redundant, too invasive and problematic for long term implementation	5, 17, 26, 28, and 29
Difficulty remembering	Forgetting to interact with telehealth system and having to be reminded	5, 17, and 23
Repetitive process	Perceiving telehealth monitoring and content to be boring or monotonous	17 and 35
Healthcare services	Barriers to telehealth relating to access or use of services delivering healthcare	
Preference for in-person care	Concern about the loss of personal contact with nurses, feeling that some services could not be delivered via telehealth, and finding face-to-face contact with healthcare professionals important	7, 27, 34, and 35

### Patient Facilitators of Telehealth

Patient facilitators of telehealth were reported in 29 studies (ESM 7: [19, 22–24, 43–61, 64–68, 70, 71, 73, 76, 77]). Nine individual facilitators were recorded: Improved self-care, increased access to healthcare, improved health knowledge, ease of use, peace of mind, convenience, effective health management, appreciation of telehealth nurses, and believing telehealth to be as good or better than in-person care (see Table 4).

**Table 4:** Facilitators of telehealth

Facilitator	Definition	Paper number
Health management	Facilitators of telehealth that relate to improved health or improved management of health condition	
Improved self-care	Empowers patients to manage their health condition better, as it makes them more careful and more concerned about their health. It allows them to play a more active role in their health management, thus leading to improvements in symptom recognition and symptom management	1, 6–13, 16, 17, 20–24, 27, 29, 32, 33, and 35
Improved health knowledge	Educates patient about their health, by providing more accurate information in smaller pieces over time, which helps to reinforce material, consequently giving patients a better understanding and awareness of their condition	4, 5, 9–11, 13, 17, 21, 23, 27, 29, and 33
Effective health management	Patients perceive telehealth to be a lifesaver, as it helps maintain health stability and leads to improvements in patient clinical outcomes. Plays a preventative role and diminishes potentially negative health outcomes	5, 10, 13, 15–17, 27, 32, and 35
Healthcare services	Facilitators of telehealth relating to access or use of services delivering healthcare	
Improved access to care	Healthcare professionals are able to review the results of patient self-testing immediately, and see any early warnings of health status deterioration, thus reducing the number of emergency department visits and hospital admissions	3, 5–7, 9, 10, 13–15, 21, 24, 27, and 32–35
Happy/confident in nurse advice	Patients receive feedback and focused motivational support on self-management from the nurses and the nurses are able to address any problems patients have	5, 6, 7, 9, 15, and 23
As good/better than in-person care	Care received through telehealth is seen to be as good as a visit from the nurse and patients prefer to take their measurements themselves at home, as they feel comfortable there	9, 13, 27, and 34
Patient variables	Facilitators relating to patients’ beliefs about the benefits of telehealth	
Convenient	Telehealth is more useful and convenient than other methods of healthcare delivery, as it takes very little time and does not interfere with usual activities. Patients also benefit from decreased traveling, time saved, and fewer medical visits	2, 5, 11, 12, 17, 21, 27, 32, and 35
Peace of mind (regarding health)	Patients feel safer and more confident when participating in telehealth monitoring. They are informed about their health status, and kept regularly aware of the results, therefore they get to know whether their body is functioning well, and do not worry about their health as much	2, 6, 9, 10, 13, 26, 27, 32, 33, and 35
Technology-related	Facilitators relating to technology, which make it easy for patients to use telehealth	
Ease of use	Find working with the telehealth equipment to be not difficult at all and verbalize that telehealth is not technologically intimidating	1, 2, 5, 11, 12, 16, 17, 20, 21, 22, 24, and 27

### Appraising the Quality of Evidence

According to the quality criteria applied, 11 studies were classified as high quality, 23 were categorized as moderate quality, and three were classified as low quality (ESM 4). One high-quality study reported a refusal rate for telehealth participants only, with 48 % declining to participate [72]. Seven moderate-quality studies reported refusal rates for telehealth participants only, with an average of 31 % (range, 4–71 %) refusing telehealth [45, 48, 50, 51, 61, 63, 65, 68]. One high-quality and four moderate-quality studies provided reasons for participant refusals [45, 50, 51, 61, 65, 72]. Demographics for refusals were provided by one high-quality and two moderate-quality studies [50, 51, 61, 72]. Six high-quality studies reported participant withdrawals from the study, with an average of 20 % (range, 4–48 %) of participants who agreed to participate subsequently withdrawing [22, 47, 69, 72–74]. Fifteen moderate-quality studies reported participant withdrawals from the study, with an average of a 23 % (range, 5–55 %) withdrawal rate [19, 43, 45, 46, 49–51, 58, 60–63, 65, 67, 75, 78, 79]. Two high-quality and 12 moderate-quality studies provided reasons for participant withdrawal [19, 22, 43, 46, 47, 49–51, 58, 60–63, 65, 67, 75]. One moderate-quality study provided demographics for withdrawals [65].

The most frequently reported barriers to telehealth acceptance in the high-quality papers were: Technical problems [47, 54, 69, 76]; and believing telehealth to be unnecessary [22, 47, 72, 73]. Among the moderate-quality papers, the most frequently reported barriers to telehealth acceptance were: Technical problems [19, 49, 56, 67, 71, 79]; and preference for in-person [19, 24, 49]. The most frequently reported facilitators of telehealth acceptance reported in the high-quality papers were: Improved self-care [22, 23, 54, 57, 66, 73, 76], improved health knowledge [22, 23, 47, 54, 57, 73], and effective health management [22, 23, 47, 54, 57, 76]. Among the moderate-quality papers, the most frequently reported facilitators of telehealth acceptance were: Improved self-care [24, 43, 48–53, 55, 56, 61, 65, 67, 68, 77] and increased access to healthcare [19, 24, 45, 48, 49, 52, 53, 60, 65, 68, 77].

## Discussion

The aims of the review were to assess the levels of uptake and sustained use of home telehealth and to identify the factors that influence whether patients with a diagnosis of HF or COPD accept or refuse and sustain using or abandon telehealth. Overall, studies reported that almost one third of the patients who were eligible to receive telehealth refused to participate and one fifth of patients who agreed to take part in the studies later withdrew their consent. Studies showed a wide range of both refusals (4–71 %) and withdrawals (4–55 %). Twenty-one studies reported participant withdrawals from the study, relating to people offered telehealth only, however only eight studies reported participant refusal rates, therefore levels reported in this review may not be reflective of the whole picture, owing to the small sample. Five out of eight studies documented reasons for refusals to participate and 14 out of 21 studies provided reasons for participant withdrawals. However, study reports did not go into great detail, meaning that caution is warranted in interpreting these reports as accurate accounts of participants’ reasons for refusing or withdrawing. Limited details were provided regarding the demographics of patients who refused to participate, or withdrew. Although 23 studies commented on participant refusals and/or withdrawals, only four studies provided some form of demographic data for these participants. From the data provided by these studies it appeared that there were very few differences in relation to the age and gender of people who refused compared with participants who accepted. However, only one study reported details about the demographics of the participants who withdrew, meaning it is difficult to draw any comparisons between the participants who accepted a telehealth intervention and those who withdrew from the intervention.

We cannot assess how the refusal and withdrawal rates in the current review compare with studies of other interventions because papers do not consistently report refusal or withdrawal rates [80]. This is problematic, because there is no benchmark rate of refusal or withdrawal against which interventions can be compared. Moreover, some reporting guidelines are not as explicit as others, for example the Standards for Quality Improvement Reporting Excellence (SQUIRE) guidelines do not state that information relating to intervention acceptance and withdrawal should be recorded [81], whereas the Consolidated Standards of Reporting Trials (CONSORT) guidelines state that authors should report losses and exclusions after randomization for each group, together with reasons [82]. Thus, to assess the generalizability and comparability of interventions, all reporting guidelines should explicitly state the need for clear and detailed reporting of participant refusals and withdrawals [83]. Furthermore, uptake and withdrawal may be impacted by the health status of the individual. For example, patients who are very unwell and those who are relatively well may not want to use telehealth because they think either that they are too ill to use it or not ill enough, and therefore refuse or abandon telehealth. Thus, papers need to report more contextual information, such as health status, so that patients can be compared with their counterparts. The majority of papers included in the review were RCTs, where patient withdrawal from interventions has been found to be lower compared with practice [84]. RCTs are generally expensive to conduct, and so patients who are believed to have the ability and motivation to complete the intervention are more likely to be selected to participate [85]. The RCT studies included in the current review may therefore underestimate levels of abandonment compared with practice, as they may have included people who were selected as being more likely to complete the trial.

The review identified seven individual patient barriers to telehealth acceptance and nine individual patient facilitators of telehealth acceptance. Barriers and facilitators were identified across all study designs; however, the majority of barriers and facilitators were identified in the qualitative or mixed methods studies. This is most likely explained by the fact that qualitative studies are better able to identify patient reported barriers and facilitators [86], as they tend to assess patient perceptions. The most frequently reported barriers to telehealth were: Technical problems, believing telehealth to be unnecessary, and a preference for in-person care. Patients find it difficult to use or understand technology and many experience technical difficulties when using telehealth [87, 88]. These complaints may be explained by the fact that according to a hospital administrator, “people have a natural resistance to change and patients are reluctant to try something new” [87]. Consequently, patients often decide to decline or quickly abandon telehealth, as a result of the technical problems that occur, or are expected to occur. Patients also deem traditional communication via face-to-face contact between nurses and patients to be more natural, free and unrestrained, as compared with telehealth interactions [87, 89]. To overcome these patient barriers to telehealth, it would be useful for healthcare professionals to ensure that patients, who are being offered telehealth, understand why they are being asked to use it and the benefits it can provide. Furthermore, the recent whole systems demonstrator evaluation study found satisfaction with telehealth equipment to be one of the main predictors of continued participation in the whole systems demonstrator trial [90]. Therefore, it would be advantageous to verify that patients are able to use the telehealth equipment competently and that any problems they encounter are resolved as quickly as possible, to minimize negative perceptions. It may also be useful for healthcare providers to offer telehealth to patients at an earlier stage, to avoid patients becoming accustomed to in-person visits from health professionals. However, both health professionals and researchers should also be aware that telehealth is not ideal for all patients and that some may never want to use telehealth or be well enough to use the equipment.

The most frequently reported facilitators of telehealth were: Improved self-care, increased access to healthcare, and improved health knowledge. Patients think telehealth enables them to better manage their health, by giving them better physiological control [91–95]. Telehealth also increases patient health knowledge, as it gives them a better understanding of their medical condition [96]. Patients also appreciate the fact that telehealth can facilitate a quick response to any health problems [65]. To improve uptake and sustained use, providers should consider communicating these facilitators of telehealth to patients. Patients should be informed that telehealth could help to educate them about their health condition, lead to improved self-care and can also provide better access to healthcare professionals.

In the current review, the quality of included studies was assessed using the Critical Appraisal Skills Program critical appraisal checklists, which address the key domains of methodological quality. Despite several studies in the current review having methodological weaknesses, there were no major differences in the reporting of participant recruitment by all of the 37 included studies, as compared with those reported by the 11 high-quality studies. Participant refusal rates were greater in the high-quality studies, as compared with the moderate-quality studies; however only one high-quality study reported the refusal rate for telehealth participants only, in comparison to seven moderate-quality studies. Thus, it could be argued that some of the studies which were regarded as being high-quality were not actually that, but rather they were written up in a high-quality way. Furthermore, almost all of the barriers and facilitators that were identified as being the most reported when including all studies regardless of methodological quality were each reported in multiple high-quality papers. Thus, it appears that the quality of the papers did not have a major effect on the reported results with respect to the barriers and facilitators of telehealth.

Healthcare professionals have a key role to play in patient recruitment to telehealth interventions. Evidence would suggest that, in research trials, frontline staff effectively screen patients for whom they consider telehealth would be beneficial [59]. For that reason, it is unknown how beneficial telehealth would be for those patients who are informally screened out, who decline it, or who drop out. Furthermore, it is unknown whether there is any influence from clinical staff over whether patients decide to accept or decline telehealth. A recent systematic review by Brewster and colleagues [97] concluded that frontline staff acceptance is essential, to implement telehealth at scale. However, previous research has found that some healthcare providers remain to be convinced about telehealth. For example, some nurses are concerned that telehealth would make them redundant and thus, they are often less enthusiastic than patients in the initial phases of telehealth implementation [35, 98–100]. Furthermore, the RCN ehealth survey 2012 report found nursing staff that had experience of telehealth were more likely to be positive about it and to have more knowledge about the benefits than staff with no experience of telehealth [101]. It would be valuable for future research to investigate whether healthcare providers’ negative perceptions of telehealth could affect patients’ decisions to accept or decline telehealth [99].

To our knowledge, this is the first systematic review to examine levels of home telehealth uptake and abandonment and the factors that influence HF and/or COPD patients’ decisions to accept, refuse, or sustain using telehealth. A potential limitation of the present review relates to the issue of publication bias. For this review, we relied on the information that was provided in published literature only, therefore, we could have missed important gray literature. However, the problem with gray literature is that some associations or organizations can publish reports and working papers that have their own political or social agendas, thus the validity of evidence may be questionable as a result of selective reporting [102]. Secondly, we excluded mobile phone-based interventions because mobile-based systems are currently not a mainstream means of delivering home telehealth. However, mobile phones are increasingly taking the place of fixed-line phones at home, and telehealth applications on mobile phones look likely to become a viable alternative for home telehealth in the future. A third limitation is that information has only been obtained from research studies, where patients may have been selected to participate on account of being more likely to engage in sustained use of telehealth. Furthermore, the refusal rates in these studies may be more representative of patients refusing to take part in a trial, rather than refusing telehealth per se. A fourth limitation is that it is difficult to differentiate between the facilitators relating to uptake or sustained use, as it is unknown whether the advantages of telehealth, specified by patients, apply to their decision to accept or sustain using telehealth.

The present review has consolidated current knowledge on the rates of uptake, refusal, and sustained use of home telehealth and has documented reasons for patient refusals and withdrawals from participation. It is evident that research needs to report more detail relating to participant refusal and withdrawal rates and their reasons for this. One explicit recommendation would be for future trials to report refusal rates for both study participants and telehealth participants. The review also identified an extensive range of barriers to, and facilitators of, telehealth. Technical problems appeared to be a major issue impacting on the uptake and sustained use of telehealth, with studies reporting little tolerance for poorly working systems, thus it is essential that telehealth equipment is user friendly and functions effectively. Furthermore, users can be unsure of the technology, hence appropriate training and access to support could also support uptake and use. Compared with quantitative studies, qualitative studies produced a wider range of barriers to and facilitators of telehealth. Therefore, further qualitative work is required [28], which will help to explain the barriers and facilitators that currently lead to patient uptake or refusal, and sustained use or abandonment, of telehealth. Future research should also aim to understand the role of clinical staff in how telehealth is offered and to consider how this could impact on uptake. This future research will then hopefully assist in the design of tools to predict which people may be likely to take up and sustain using telehealth and those for whom it would not be appropriate.

## Electronic Supplementary Material

Below is the link to the electronic supplementary material.

ESM 1 (DOC 316 kb)
