# Supplementary material for: Home Telehealth Uptake and Continued Use Among Heart Failure and Chronic Obstructive Pulmonary Disease Patients: a Systematic Review
Source: Ann Behav Med. 2014 Apr 25;48(3):323–36. doi: 10.1007/s12160-014-9607-x (PMC4223578; doi:10.1007/s12160-014-9607-x)
Supplement: Supplementary file 1 — (DOC 316 kb) [file 12160_2014_9607_MOESM1_ESM.doc]

**Electronic Supplementary Material 1.** Data Extraction Sheet

Source

Study ID:

Article title:

Publication:

Country data collected:

Eligibility

Eligibility for review:

Study Design

Study design:

Sample recruitment:

Recruitment context:

Aim:

Focus of paper:

Study Characteristics

Inclusion criteria:

Exclusion criteria:

Total study duration:

Total number of participants:

Diagnostic criteria:

Age:

Sex:

Socio-demographics:

Usual care:

Interventions

Description of the intervention:

Input from health professional:

Theoretical basis for intervention:

Outcome Data

Number of participants included in analysis:

Number of refusals:

Reason for refusals:

Number of withdrawals, exclusions, lost to follow-up:

Reason for withdrawals:

Refusal/withdrawal demographics:

Length of follow-up, number and/or times of follow-up measurements:

Outcome variable:

Results of TH implementation:

Positive perceptions of telehealth:

Negative perceptions of telehealth:

Unit of assessment/analysis:

Summary outcome:

**Electronic Supplementary Material 2.** Narrative Synthesis Framework

| **Main elements of synthesis** | **Review** |
| --- | --- |
| 1. Developing a theory of how the intervention works, why and for whom | - Inform decisions about the review question and what types of studies to review - Contribute to the interpretation of the reviews findings - Assess the applicability of the findings |
| 1. Developing a preliminary synthesis of findings of included studies | - Organise and describe findings through textual descriptions/thematic analysis to provide an initial description of patterns across the included studies - Identify and list the reported facilitators and barriers to telehealth adoption - Explore the relationship between reported facilitators and barriers |
| 1. Exploring relationships in the data | - Rigorously scrutinise patterns which emerged from the data in the preliminary synthesis in order to identify factors that may explain variations in the facilitators and/or barriers to successful telehealth adoption - Explore relationships between study results and the key aspects of study population, intervention and context - Explore relationships between the findings across different studies |
| 1. Assessing robustness of synthesis | - Assess the strength of the evidence for drawing conclusions about the facilitators and/or barriers to telehealth adoption identified in the synthesis - Assess the generalisability of the product of the synthesis to different population groups and/or contexts |

| **Paper** | **Reason for Exclusion** |
| --- | --- |
| Antonicelli et al. (2008) | No details on patient acceptance, abandonment or perceptions |
| Bourbeau et al. (2003) | Acute exacerbations of COPD |
| Boyne et al. (2013) | No intervention |
| Cardozo & Steinberg (2010) | Data/results for HF/COPD not presented in isolation |
| Cawley et al. (2011) | No intervention |
| Chandler (1990) | Data/results for HF/COPD not presented in isolation |
| Chau et al. (2012) | Technology used mobile phone |
| Chaudhry et al. (2010) | No details on patient acceptance, abandonment or perceptions |
| Cummings (2010) | Acute exacerbations of COPD |
| Dale et al. (2003) | No details on patient acceptance, abandonment or perceptions |
| Delaronde (2002) | No details on patient acceptance, abandonment or perceptions |
| Demiris et al. (2003) | Data/results for HF/COPD not presented in isolation |
| de Lusignan et al. (1999) | Data presented in full in de Lusignan (2000) |
| De Toledo et al. (2006) | No details on patient acceptance, abandonment or perceptions |
| Dougherty et al. (2005) | Data/results for HF/COPD not presented in isolation |
| Elwyn et al. (2012) | No patient input |
| Finklestein et al. (2004) | Data/results for HF/COPD not presented in isolation |
| Finkelstein et al. (2006) | Data/results for HF/COPD not presented in isolation |
| Finkelstein et al. (2010) | No details on patient acceptance, abandonment or perceptions |
| Hicks et al. (2009) | Data/results for HF/COPD not presented in isolation |
| Hill et al. (2008) | Technology not used to deliver healthcare |
| Hoover et al. (2007) | No details on patient acceptance, abandonment or perceptions |
| Hopp et al. (2006) | Data/results for HF/COPD not presented in isolation |
| Jensen et al. (2012) | No details on patient acceptance, abandonment or perceptions |
| Johnston et al. (2000) | Data/results for HF/COPD not presented in isolation |
| LaFramboise et al. (2003) | No details on patient acceptance, abandonment or perceptions |
| Liddy et al. (2008) | Data/results for HF/COPD not presented in isolation |
| Lind et al. (2013) | Limited details on patient perceptions |
| Mair et al. (2002) | Hospitalized patients |
| Marno et al. (2010) | Technology not used to deliver healthcare |
| Marzegalli et al. (2008) | No HF/COPD patients |
| Marziali (2009) | No HF/COPD patients |
| Masella et al. (2008) | Hospitalized patients |
| Merilahti et al. (2009) | No HF/COPD patients |
| Miravitlles et al. (2002) | Acute onset of COPD |
| Morguet et al. (2008) | Not all patients aged > 18 |
| Pare et al. (2006) | No details on patient acceptance, abandonment or perceptions |
| Partridge (2004) | Data/results for HF/COPD not presented in isolation |
| Pecina et al. (2011) | Data/results for HF/COPD not presented in isolation |
| Peikes et al. (2009) | Data/results for HF/COPD not presented in isolation |
| Pinna et al. (2003) | Data presented in Pinna (2007) |
| Piotrowicz et al. (2010) | Technology used mobile phone |
| Prescher et al. (2013) | Technology used mobile phone |
| Rogers & Schott (2008) | No intervention |
| Rosenman et al. (2006) | Data/results for HF not presented in isolation |
| Sanders et al. (2012) | Data/results for HF/COPD not presented in isolation |
| Schou et al. (2013) | Acute exacerbation of COPD |
| Sciacqua et al. (2009) | Limited details on patient perceptions |
| Seto et al. (2011) | Technology not used to deliver healthcare |
| Shea & Chamoff (2012) | No details on patient acceptance, abandonment or perceptions |
| Sicotte et al. (2011) | Acute exacerbations of COPD |
| Sorknaes et al. (2011) | Acute exacerbations of COPD |
| Subramanian et al. (2004) | No HF/COPD patients |
| Terschuren et al. (2012) | No HF/COPD patients |
| Vitacca et al. (2009) | Data/results for COPD not presented in isolation |
| Vontetsianos et al. (2005) | Limited details on patient perceptions |
| Wakefield et al. (2008) | Limited details on patient perceptions |
| Zickmund et al. (2008) | No HF/COPD patients |

**Electronic Supplementary Material 3.** Full text papers that were reviewed but excluded

**Electronic Supplementary Material 4.** Details of the Included Studies

| **Paper/ report authors** | **Location of trial/ initiative** | **Type of trial/ initiative** | **Focus of Paper** | **Recruitment Context** | **Description of intervention** | **Input from health professional** | **Patient Diagnosis** | **Sample Size** | | | **Quality** |
| --- | --- | --- | --- | --- | --- | --- | --- | --- | --- | --- | --- |
|  |  |  |  |  |  |  |  | **Total** | **TG** | **CG** |  |
| 1. Antoniades et al. (2012) | Australia | RCT | Feasibility and effectiveness | Metropolitan tertiary-care hospital | Remote monitoring and transmission of physiological data | Reviewed data to detect deterioration and to determine need for intervention | COPD | 44 | 22 | 22 | Moderate |
| 2. Bedra et al. (2013) | USA | Qualitative | Feasibility and acceptance | Johns Hopkins Bayview  Medical Center | Pulmonary telerehabilitation system | N/A – system was demonstrated to patients | COPD | 21 | 21 | N/A | Low |
| 3. Bowles et al. (2011) | USA | RCT | Effectiveness | Philadelphia area hospitals | Remote monitoring and transmission of physiological data, and intermittent video visits | Monitored data for out of range readings and conducted and video visits | HF | 218 | 102 | 116 | Moderate |
| 4. Casas et al. (2006); Garcia-Aymerich et al. (2007) | Spain, Belgium | RCT | Effectiveness | Two tertiary hospitals (Barcelona, Leuven) | Physical and social assessment, and education | Reinforced self-management strategies and inquired about use of healthcare resources | COPD | 155 | 65 | 90 | Moderate |
| 5. Clark et al. (2007) | Australia | Mixed method | Adherence, adaptation and acceptance | Sample taken from participants who completed Assistance by Telephone study | Nurse coordinated telephone-monitoring support, health questions, and education | Coordinated telephone-monitoring support and provided education materials | HF | 79 | 79 | N/A | High |
| 6. Delaney & Apostolidis (2010) | USA | Cohort | Feasibility | Multibranch Medicare certified non-profit home care agency | Remote monitoring and transmission of physiological data, evidence-based education, HF assessment, and therapeutic activities | Reviewed data and responded by calling patient and notifying physician if indicated with any abnormal findings | HF | 24 | 24 | N/A | Moderate |
| 7. de Lusignan et al. (2001) | UK | RCT | Acceptability, effectiveness and reliability | General Practice database | Remote monitoring and transmission of physiological data, and video consultation | Reviewed data collected | HF | 20 | 10 | 10 | Moderate |
| 8. Domingo et al. (2011; 2012) | Spain | Cohort | Effectiveness and acceptance | Multidisciplinary HF unit in a university hospital | Remote monitoring and transmission of physiological data, educational videos, motivational messages, and questionnaires | Sent information, provided educational videos and reviewed data | HF | 97 | 97 | N/A | Moderate |
| 9. Fairbrother et al. (2012; 2013a); Pinnock et al. (2012) | UK | Mixed method | Effectiveness and patient perceptions | Hospital and community-based specialist respiratory services’ records | Remote monitoring and transmission of physiological data, symptoms and use of medication | Reviewed data and contacted patients if questionnaire responses and physiological data fell outside expected range | COPD | 256 | 128 | 128 | Moderate |
| 10. Fairbrother et al. (2013b) | UK | Qualitative | Patient perceptions | Postal invitations to patients involved with the telemonitoring service in Lothian, Scotland | Remote monitoring and transmission of physiological data and educational content | Monitoring of data and initiation of contact with patients to address any matters arising related to transmitted data | HF | 18 | 18 | N/A | High |
| 11. Finkelstein et al. (2010) | USA | Mixed method | Feasibility | Not reported | Remote monitoring and transmission of physiological data, health questions, support in following individualized treatment plans, and education | Reviewed patient data, and generated individualized alerts and action plans for each patient whenever warranted | HF | 10 | 10 | N/A | Moderate |
| 12. Finkelstein & Wood (2011) | USA | Mixed method | Feasibility | Not reported | Remote monitoring and transmission of physiological data, education and counseling, individualized treatment plan, guideline-concordant decision support | Reviewed patient data, tracked progress, made changes to medications, and set alerts | HF | 10 | 10 | N/A | Moderate |
| 13. Gale & Sultan (2013) | UK | Qualitative | Patient perceptions | Sandwell Community Respiratory Service | Remote monitoring and transmission of physiological data | Reviewed data, triaged the readings and took appropriate action | COPD | 7 | 7 | N/A | High |
| 14. Johnston & Weatherburn (2010) | UK | Qualitative | Perceptions | Patients participating in research trial were interviewed | Remote monitoring and transmission of physiological data | Monitored data against pre-defined limits | HF | 14 | 8 | 6 | Low |
| 15. Kim et al. (2012) | Korea | RCT | Effectiveness | Hospital’s respiratory internal medicine department | Remote monitoring and transmission of physiological data and teleconsultation services | Monitor patient status remotely, educate patients on drug administration and self-management and provide consultations | COPD | 144 | 144 | N/A | Moderate |
| 16. Kulshreshtha et al. (2010) | USA | RCT | Effectiveness | Massachusetts General Hospital | Remote monitoring and transmission of physiological data | Monitored data, and offered timely interventions and teaching | HF | 110 | 42 | 68 | Moderate |
| 17. LaFramboise et al. (2009) | USA | Qualitative | Perceptions | Drawn from parent study | Health questions, education, and disease management | Viewed patient responses and determined the need for intervention | HF | 105 | 105 | N/A | High |
| 18. Lewis et al.(2010) | UK | RCT | Effectiveness | Chronic disease management team pulmonary rehabilitation database | Remote monitoring and transmission of physiological data | Monitored data and received alerting email messages if certain conditions were detected | COPD | 40 | 20 | 20 | Moderate |
| 19. Louis et al. (2003) | UK | RCT | Acceptance | Not reported | Remote monitoring and transmission of physiological data | Not reported | HF | 420 | 162 | 258 | Moderate |
| 20. Lovell et al. (2002) | USA | Mixed method | Effectiveness and patient perceptions | Not reported | Remote monitoring and transmission of physiological data | Managed patient  data and controlled scheduling of measurements | HF/ COPD | 22 | 22 | N/A | Low |
| 21. Maric et al. (2010) | Canada | Cohort | Effectiveness | Heart Function Clinic | Remote monitoring and transmission of physiological data, health questions, and reinforcement of self-management | Monitored patient health status and reinforced self-monitoring skills | HF | 20 | 20 | N/A | Moderate |
| 22. Nahm et al. (2008) | USA | Qualitative | Acceptance | Pool of enrollees in Medicare Coordinated Care Demonstration project | Remote monitoring and transmission of physiological data, and interactive Webbased learning modules | N/A –Web learning module and telemonitoring  devices were demonstrated to patients | HF | 44 | 44 | N/A | High |
| 23. Nguyen et al. (2008) | USA | RCT | Effectiveness | Recruitment announcements were sent to email distribution lists and online COPD support groups | Self-monitoring of exercise and respiratory symptoms and reinforcement of dyspnea management strategies, structured education, skills training, and peer interactions | Dyspnea and exercise consultation, reinforcement telephone calls/emails, group sessions on management | COPD | 50 | 26 | 24 | Moderate |
| 24. Piette et al. (2008) | USA | Cohort | Feasibility | Electronic medical records in university based healthcare system | Automated telephonic assessment and behavior change service | Received notification when a patient reported an urgent medical condition | HF | 52 | 52 | N/A | Moderate |
| 25. Pinna et al. (2007) | Italy, Poland, UK | RCT | Effectiveness | HF patients enrolled in 11 hospitals from 3 different European countries | Remote monitoring and transmission of physiological data, and non-invasive cardio-respiratory and activity monitoring | Provided telephone assistance and managed and recorded patient data | HF | 195 | 195 | N/A | High |
| 26. Radhakrishnan et al. (2012) | USA | Mixed method | Patient perceptions | Flyers distributed to home care agency telehealth patients | Remote monitoring and transmission of physiological data | Reviewed patient data and followed up with patient if necessary | HF | 4 | 4 | N/A | Moderate |
| 27. Rahimpour et al. (2008) | Australia | Qualitative | Patient perceptions | Databases from Prince of Wales Hospital, Sydney | Remote monitoring and transmission of physiological data, medication reminders and measurement scheduling | N/A – video demonstration of system was shown to patients | HF/ COPD | 77 | 77 | N/A | High |
| 28. Schmidt et al. (2008) | Germany | Cohort | Effectiveness and acceptance | Ambulatory heart failure clinics | Medication box connected to electronic health record via signal transmissions of a microprocessor | Monitored electronic health record and initiated interventions if necessary | HF | 62 | 32 | 30 | High |
| 29. Seibert et al. (2008) | USA | RCT | Effectiveness | Not reported | Remote monitoring of physiological data and health questions | Contacted patients to conduct a chart review | HF | 23 | 13 | 10 | High |
| 30. Spaeder et al. (2006) | USA | RCT | Effectiveness | Johns Hopkins Hospital and Medical Center | Remote monitoring and transmission of physiological data, and health questions | Reviewed patient data | HF | 49 | 25 | 24 | High |
| 31. Trappenburg et al. (2008) | Netherlands | Cohort | Effectiveness | Hospitals in the center of the Netherlands | Remote monitoring and transmission of physiological data; health questions, and education | Reviewed patient answers and data, and contacted patient if values were alarming | COPD | 115 | 59 | 56 | Moderate |
| 32. Ure et al. (2012) | UK | Qualitative | Feasibility and acceptance | NHS Lothian pilot patients were invited to participate in qualitative interviews | Remote monitoring and transmission of physiological data | Contacted patient or primary care practice according to algorithm based on questionnaire responses | COPD | 20 | 20 | N/A | High |
| 33. Venter et al. (2012) | New Zealand | Mixed method | Acceptance and utilization | Not reported | Remote monitoring and transmission of physiological data | Monitored patient record and contacted patients for clinical intervention | HF/ COPD | 20 | 10 | 10 | Moderate |
| 34. Whitten & Mickus (2007) | USA | Mixed method | Patient health outcomes and attitude | Marquette General Health System | Remote monitoring and transmission of physiological data, and videoconferencing | Provided real-time video visits | HF/ COPD | 161 | 83 | 78 | Moderate |
| 35. Whitten et al. (2009) | USA | Mixed method | Effectiveness and perceptions | St. Vincent's Heart Failure Unit | Remote monitoring and transmission of physiological data | Conducted home health visit, and telephone interviews | HF | 50 | 50 | N/A | Moderate |
| 36. Wong et al. (2005) | China | RCT | Effectiveness | Acute care hospital in Hong Kong | Educational and supportive telephone follow-up programme | Conducted telephone follow-up | COPD | 60 | 30 | 30 | Moderate |
| 37. Wu et al. (2005) | Canada | Cohort | Feasibility and patient acceptance | Heart Function Clinic | Remote monitoring and transmission of physiological data | Reviewed and responded to patients' entries | HF | 62 | 62 | N/A | Moderate |

*TG = Telehealth group

*CG = Control group

*HF = Heart Failure

*COPD = Chronic Obstructive Pulmonary Disease

**Electronic Supplementary Material 5.** Study design definitions

| **Study design** | **Definition** |
| --- | --- |
| Randomised Controlled Trial (RCT) | An RCT is an experimental study where participants (individuals or groups) are randomised either to receive the intervention being tested or to receive a control treatment (usually the standard treatment or a placebo). |
| Mixed method | Mixed methods studies involve a combination of quantitative and qualitative research methods. For example, in a mixed methods study the quantitative aspect may look at the effect of an intervention and the qualitative aspect may look at participant perceptions of the intervention. |
| Qualitative | Studies which look at the experiences, behavior or views of individuals and groups. |
| Cohort studies | A study in which a defined group of people (the cohort) is followed over time, to examine associations between different interventions received and subsequent outcomes. A ‘prospective’ cohort study recruits participants before any intervention and follows them into the future. A ‘retrospective’ cohort study identifies subjects from past records describing the interventions received and follows them from the time of those records. |

**Electronic Supplementary Material 6.** Barriers to Telehealth, Source and Text

| **Barrier** | **Source** | **Text** |
| --- | --- | --- |
| Technology-related | | |
| Technical problems | Bedra (2) | Slightly complicated to use the telecare device  Slightly difficult to use the keypad |
|  | Clark (8) | Difficulty connecting to the system  Multiple attempts to get connected  Inability to connect to the system because the system was down  Patient difficulty connecting to the system  Technical difficulties  Technical failure  Patient difficulties using the system  CHAT system failure |
|  | de Lusignan (8) | Equipment failure (3)  Battery failure (3)  Loss of data in the device memory  Constant buzz on telephone line |
|  | Fairbrother 2013b (6) | Numerous technical difficulties with the technology  Experiencing technical problems with the equipment  Recurrent malfunctions with the peripheral devices  Intrusiveness of the equipment noise and luminosity  High frequency of equipment failure reported by patients  Need for improved technology |
|  | Finkelstein 2011 (2) | I don’t like it when it doesn’t fit the screen and I have to scroll  I don’t like the controller stick |
|  | Johnston (3) | Installation of the equipment was the most common problem  Required assistance with installation  Weighing procedure presented a problem |
|  | Nguyen (15) | Technical and usability challenges (3)  Difficulties accessing Web application  Technical issues with access to study website  Decreased accessibility  Slow loading of the Web application  Usability challenges with wireless-enabled PDA  PDA didn’t allow document of data when left the city  Technical glitches need to be fixed  Technical problems decreased participant engagement  Considerable technical and usability challenges  Technical challenges accessing web and using PDA  Unreliable wireless coverage  Inconsistent wireless coverage was problematic |
|  | Pinna (11) | Non-practicable transmissions (2)  Failed transmissions  Transmission of NICRAM recordings failed  Unreliable transmission of NICRAM recordings  Failures of system  Technical problems (3)  Technical difficulties  Technical limitations of recording device |
|  | Radhakrishnan (2) | Inability of the telehealth pulse oximeters to read oxygen saturation  Equipment malfunction |
|  | Ure (8) | Irritations with the technology  The size and background noise of the computer fan caused some problems  System failed to confirm that data had been transmitted  Technical failures were a recurring concern  Battery failure in one of the peripherals  Series of technical hitches  Lack of confirmation of data transmission  Unfriendly professional interface potentially compromised communication |
|  | Whitten 2007 (2) | Data issues  Suffered data loss during collection |
|  | Wu (4) | Occurrences when the system was not available  System downtimes of several days  Several server crashes  Software problems |
| Technical anxiety | Radhakrishnan (8) | Terminated the use of telehealth due to increased anxiety related to telehealth usage  Challenging to some patients and caused anxiety and annoyance  Anxious about performing the telehealth procedure in the morning  Anxiety for elderly  Increased patient and spousal anxiety due to telehealth usage  Equipment problems resulted in anxiety, annoyance, and disenchantment  Causative anxiety  Anxious personality |
|  | Rahimpour (7) | Feared and avoided to be confronted with modern technology, eg. HTMS  Freeze up when confronted with system, complicated, something feared  Perceived system as a computer and expressed computer anxiety  The system is very hard to use  Don’t know what will happen to computer if I press the wrong button  Concerns on issue of anxiety related to the use of the HTMS  Reported fear of using system |
|  | Whitten 2007 (2) | Anxiety about working technology  Hesitancy about using technology |
| Technical support | Nguyen (3) | Participants had to install proprietary security software  Required remote assistance from technical support  Technical support before access to site |
|  | Rahimpour (3) | Concerned about technical support and maintaining the system  System needs to be regularly maintained to make sure it works properly  If it needs repair, who is responsible for that |
|  | | |
| Telehealth process | | |
| Difficulty remembering to interact with system | Clark (2) | Often forgot to ring  Reminders to call-in |
|  | LaFramboise (2) | Difficult to remember to interact with the Health Buddy daily  Difficult to remember to do it |
|  | Nguyen (2) | Never remembered goal setting or graphing on web  Lapses in exercise entries |
| Repetitive process | LaFramboise (5) | Perceived content to be boring or monotonous  So repetitious  Same thing every day, got kind of bored with it  Didn’t see the point of it, it seemed kind of foolish  Questions were intentionally repetitious |
|  | Whitten 2009 (2) | Repetition  Monotonous processes |
| Believed telehealth to be unnecessary | Clark (2) | Not much added than current care from doctor  Didn't quite get the purpose |
|  | LaFramboise (3) | Some believed 6-month time frame was too long  Less amount of time in home, and it would do the same amount of good  At 6-months was ready for it to go |
|  | Radhakrishnan (3) | Intrusive  Compromised safety  Lack of perceived usefulness |
|  | Schmidt (6) | Unnecessary to continue monitoring after study  Considered continuous control as a problem  Not needed once accustomed to medication scheme  Believed monitoring is only effective as interim tool  Too “invasive” as long-term implementation  Did not agree to continuous monitoring |
|  | Seibert (2) | 25% did not wish to continue  Redundant questions |
|  | | |
| Healthcare services | | |
| Prefer in-person care | de Lusignan (3) | Prefer to see the nurse face to face  Patients would have rather seen nurse face-to-face  Lack of additional benefit from videophone over conventional contact |
|  | Rahimpour (7) | Lack of physical presence of a healthcare provider  Physical presence of healthcare provider and face-to-face visits were essential  Face-to-face communication with your doctor is important  Importance of benefits of face-to-face visits with medical doctor  Would be better to see the doctor or nurse at home  Nothing should stop you from seeing your doctors  HTMS would not be useful for people who live near healthcare services |
|  | Whitten 2007 (4) | Loss of personal contact with nurses  Felt services best delivered in person  Felt medication help was best in person  Variety of services that could not be delivered via telehealth services |
|  | Whitten 2009 (3) | Undecided benefits versus in-person  Some tasks best in-person  Favored in-person visit |

**Electronic Supplementary Material 7.** Facilitators to Telehealth, Source and Text

| **Facilitator** | **Source** | **Text** |
| --- | --- | --- |
| Health Management | | |
| Improved self-care | Antoniades (2) | System helped them manage their COPD better (2) |
|  | Delaney (6) | Assisted them in developing a self-care schedule  Monitor has helped me get a routine. It rings and then I do the monitor and take my meds  Patient describes his readiness to self-manage his heart failure  I feel I will be able to manage it on my own.  I know what I have to do to keep things under control  Increased patients’ perceptions of knowledge regarding heart failure self-management |
|  | de Lusignan (2) | Complied better with medication  Felt devices would alert healthcare professionals to non-compliance |
|  | Domingo 2012 (5) | Positive changes in patients' behavior were observed  Improvement in behavior towards self-monitoring of weight  Positive impact on patient behavior towards managing their illness  Showed positive changes in behavior  Positive changes in patients' self-monitoring behavior and keeping a daily record |
|  | Fairbrother 2013a (11) | Increased knowledge of condition  Reinforced their decisions to adjust treatment or seek professional advice  Beneficial in determining their state of health and recognizing illness  Helpful to learn their ‘normal’ range by identifying telemonitoring data trends over time  Used measurements to inform decisions about their capacity to undertake domestic activities  If you have a bad reading you know you need to just take it easy  Used data to validate decision to self-medicate and/or to contact healthcare professionals  I can say I do need a doctor or I do need to start these steroids  Determining state of health was empowering  It gives me a lot more independence  Justified decisions to adjust treatment or seek professional advice |
|  | Fairbrother 2013b (8) | Helpful to have facility to monitor data trends over time  Beneficial in determining state of health  It keeps you in the picture  You know exactly what’s going on from day to day  Supported existing efforts to monitor weight and blood pressure  Happy to be involved  Compliant in routine monitoring behaviors  Started to realize that when felt unwell I was able to take another tablet |
|  | Finkelstein 2010 (2) | Increases self-management  Participants would review the test results once a week |
|  | Finkelstein 2011 (4) | It is helpful to record your symptoms  Expressed confidence in the utility of such intervention for their daily self-management  Expressed interest in using such a platform for self-management in the future  Useful for realizing how I feel today, it asked very relevant questions for my illness |
|  | Gale (13) | Confidence to self-manage their condition  Increased confidence to self-manage their condition (4)  Feeling more confident about their ability to manage their condition  It’s made me more confident in myself because I know what’s happening  It puts you aware of what’s happening  I’ve got my confidence back  I think it’s more or less, give me more confidence, like, knowing how I was reacting  Participants’ embodied experience of their condition, building confidence  Enabled more proactive management of the condition  Improves management of their condition |
|  | Kulshreshtha (3) | Made them feel more in control of their health  Excellent opportunity to become more aware of my disease condition  With the program I have a tendency to be diligent about my diet and weight |
|  | LaFramboise (22) | Daily reminders promoted adherence to a HF management routine  You are automatically tuned in  Kind of a ritual  It gets to be a habit, and you don’t have to worry about it, you just do it  Participants motivated to follow prescribed regimen  Effective for self-management  Motivation  Motivated to monitor and manage their heart failure  Prompted to be more active in self-management of heart failure  Teaches you the main things of how to take care of yourself  Motivated me and got me back on my feet  Taught patients how to take care of themselves  Motivates you to take better care of heart problem  Didn’t follow HF management guidelines as closely after HB removal  Health Buddy makes you more careful about what you are doing  Become less worried about your weight, after the HB is removed  Don’t follow HF routine as well, without health buddy  Effective for self-management  HB enhanced self-management abilities  Daily questions guided in understanding vigilance for self-management  Teaching and self-management provided a means to improve health  Telehealth improves symptom recognition and symptom management |
|  | Lovell (3) | Impact on improving management of chronic disease  Play an important role in managing health  More control over managing health |
|  | Maric (4) | Web site use was associated with improved self-monitoring skills  Now more concerned with what body is actually doing  Better able to maintain self-management behaviors  Better able to more confidently adhere to self-management behaviors |
|  | Nahm (2) | Research findings, medication, laboratory tests help manage HF at home  Wanted information on research findings, medications, laboratory tests |
|  | Nguyen (4) | Improved their self-efficacy for managing dyspnea  Increases in self-efficacy for managing dyspnea  Desired to “stay accountable to something or someone”  Sense of accountability and commitment |
|  | Piette (5) | Felt more confident in managing HF self-care  Make changes in self-management as a result of the assessments and follow-up  I found the system helpful for managing my heart failure symptoms  The system helped me learn how to better manage my heart failure  I learned something new about how to take care of myself from the system |
|  | Rahimpour (13) | Empower patients to manage their health conditions better  Helps to manage my condition much better  Play a more active role in their health management  Empowering patients to participate in their health management  Promotes active participation in health management  Empowered to perform better self-care, access to useful information  Empower patients to improve their self-care  Play more active role in their management  Desire to participate in their health management  The reminder system was appreciated, improved patient compliance  System can help me remember to take medications on time  Improve patients’ compliance  Improve compliance with medication and treatment |
|  | Seibert (7) | Helped them be aware of factors for managing their disease  Helped them to control and be more aware of their weight  Telemedicine unit facilitated self-care  Improved self-care  Will help them take better care of themselves in future  Facilitation of self-care  Identifying important symptoms |
|  | Ure (3) | Gave them confidence to respond to deteriorating symptoms themselves  Empowered patients to take responsibility  The machine does help you acknowledge that there’s something |
|  | Venter (2) | Easy access to TH monitoring facilitated self-management  Improved ability to manage their conditions |
|  | Whitten 2009 (10) | Allowed for increased independence  Helped to feel personally empowered over own health condition  Getting checked every day allowed you to notice and track own health  Facilitated empowerment  Sense of control over their health  Reported better management of oral medications  Telehealth visits kept them on a consistent routine  Kept them on a regular schedule  Helped establish a personal health monitoring routine  Pattern of getting used to doing it daily |
| Improved health knowledge | Clark (2) | Fluid education and other advice helped a lot  Gained a lot of information and help with aspects I didn't understand |
|  | Fairbrother2013a (6) | Support greater understanding of their COPD  Know a lot more about it [COPD] now  I knew it was a breathing problem. And I know what causes it  Now I know it’s a progressive illness, it goes in different stages  First time, they had access to clinical data about their condition  Improved understanding of COPD |
|  | Fairbrother 2013b (3) | Felt better informed and more knowledgeable about their condition  Enhanced patients’ knowledge and understanding of their condition  Found it helpful to know their weight, blood pressure and oxygen saturation score |
|  | Finkelstein 2010 (3) | Educating patients on their condition  Increase condition awareness  System supports patients in following HF action plans |
|  | Gale (6) | My oxygen levels today were 95, but were 92 two days ago, 95 is really good, 92’s good  Without this, I’m going to think, what’s my oxygen levels? Have they gone down or up?  You wouldn’t know they’d dropped unless you take the readings  Increased (medicalized) knowledge and understanding of their condition  Able to understand what it’s all about, blood pressure, SBO2s, temperature and weight  Understanding of how well their body was functioning on a day-by-day basis |
|  | Garcia-Aymerich (2) | Improved disease knowledge  Helped COPD patients to enhance knowledge |
|  | LaFramboise (13) | Daily information about HF made information easier to comprehend  Health Buddy gives health information in smaller pieces over time  Repetition reinforced educational material  Solidified participant understands of disease process and its management  HB content consistent with what participants’ physicians were telling them  Small bits of repeated information enhanced comprehension  Health Buddy helped to better understand health information  Heart failure knowledge would be improved  HB taught about HF and promoted comprehension and self-management  HB information improved their knowledge of heart failure  HB helped to clarify information previously provided by physicians  Information and repetition made comprehension more understandable  Delivers information in smaller, frequent, manageable pieces |
|  | Maric (3) | Web site made them aware of their symptoms  More aware of whether heart is bothering me or if it’s something else  Made you understand to look for any of the symptoms |
|  | Nguyen (2) | Small improvements in knowledge of dyspnea management strategies  Positive impact on perception of dyspnea |
|  | Rahimpour (7) | Become more aware of health conditions  Would improve their knowledge about their health problems  Learn about your disease and how to manage it  Clarifies many questions in your mind  Provided more accurate and more frequent info about health conditions  Accurate information about our conditions helps a lot  Improves patients’ knowledge |
|  | Seibert (2) | Better understanding their conditions  Unit helped them better understand their condition |
|  | Venter (3) | A deeper understanding of the disease and quick feedback  Gained insight into how aspects of their life affected their condition  Gained insight into the relationship between medication doses and clinical measurements |
| Effective health management | Clark (4) | Fluid and diet management  Excellent maintained and controlled my weight  CHAT helped to maintain stability always spoke highly to doctor about you  I lost 6 kg |
|  | Fairbrother 2013b (3) | Changes to medication resulting from telemonitoring  Telemonitoring was seen as a lifeline  Enhanced quality of patient care. |
|  | Gale (7) | A boon! A god-send!  It’s made the world of difference to me  Before telehealth I never knew whether I needed them  Before telehealth it was get up to A&E and admitted to hospital  If I’d had the equipment then, I’d have picked it up  I can see the early warning  If I didn’t have that [telehealth], I wouldn’t know how low I was |
|  | Kim (2) | Provided significant clinical benefits  Reduced number of home visits and hospitalizations |
|  | Kulshreshtha (2) | The program improved their HF control  The program helped them stay out of hospital |
|  | LaFramboise (17) | Effective for management of heart failure  A lifesaver  Heart failure management would be improved  Health Buddy literally saved their lives  Significantly diminished potentially negative outcomes of heart failure  Perceived without Health Buddy they likely would not be here today  HB guidance significantly decreased potential HF complications  Wouldn’t be here today if it wasn’t for HB, set me on the right path again  If I didn’t have the Health Buddy, I’d probably be dead  I called it my life line  Health Buddy prevented a heart attack  Participants perceived HB be a lifesaver  Physicians predicted significant, negative outcomes that did not occur  Participants credited HB for avoidance of negative outcomes  Effectiveness for management  HB gave directions for action if experiencing a high-risk symptom  It helped me all the way around. I mean I lost weight and everything else |
|  | Rahimpour (20) | Playing a preventative role  Providing early warning when health conditions were deteriorating  If not feeling too well, you can go back on and recheck your status  Could warn patients at an early stage of health deterioration  Provided feedback and awareness of current health conditions  Providing accurate and up-to-date health information  Able to get proper measurements more often and see how I am going  Early warning of health status deterioration  Inform patients about health status more often and more accurately  Know whether own body is functioning well from daily measurements  Informed about health status, and regularly aware of the results  Reduce number of emergency department visits and hospital admissions  Provided early warning of health status deterioration  Possible to know problem at beginning and avoid emergency  Reduce number of medical practitioner visits  Don’t have to go (to the doctor) frequently  Reducing number of medical and ED visits and hospital admissions  May reduce the use of emergency services and hospital admissions  Decrease the number of medical visits  Decrease in emergency department utilization and hospital admissions |
|  | Ure (5) | Earlier recognition of exacerbations  Technology able to detect early signs of an exacerbation  Machine can tell patient is ill even before he knows it himself  Objective measure which could distinguish those symptoms which needed clinical attention  Validated the decision to seek help |
|  | Whitten 2009 (18) | Improved ability to engage in activities  More energy at the end of the project  Reported significant improvement across an array of areas  Improvements made in patient clinical outcomes  Improvements in shortness of breath, management medications  Decreased complications with swelling in the legs and ankles  Needed to sit down and rest less frequently throughout the day  Walking or climbing the stairs became less of a challenge  Experienced fewer incidents of shortness of breath  Less likely to be tired, fatigued, or low on energy  Decreased need for hospitalization  Experienced fewer treatment side effects  Enhanced mobility  Increased sense of energy  Physical, behavioral, and emotional improvements  Experienced positive symptom changes  Provided in-depth and detailed information  Got much more consistent evaluations from the machines |
|  | | |
| Healthcare Services | | |
| Increased access to care | Bowles (2) | Expressed satisfaction in areas associated with access to care  Felt better prepared for how to contact their nurse |
|  | Clark (2) | Improved accessibility to specialist care  CHAT team organized deafness aids and phone so I have ongoing benefits |
|  | Delaney (2) | I know the nurse will call if something is wrong  If I have gained weight, I always knew she [nurse] would call |
|  | de Lusignan (2) | Telemedicine makes it easier to get medical care  Reassuring to patient know that they had ready access to help |
|  | Fairbrother 2012;2013a (11) | If anything goes wrong, you can get in touch with them any time you want  Frequency of interaction between professional and patient  If I need anything at all I've just to phone up ... it's a good service  I've got somebody feeding back and talking to me  Greater accessibility  Accessibility of telemonitoring service  Increased accessibility of telemonitoring service  Accessibility (4) |
|  | Fairbrother 2013b (6) | Continuous practitioner surveillance and support  Lets telemonitoring nurse know exactly what’s going on  If something wrong, they are going to pick it up right away  If something goes wrong, they’ll phone me  Constant practitioner surveillance (2) |
|  | Gale (21) | Legitimizing contact with health care professionals (5)  The contact patients did have was more responsive and appropriate  Nurses telephoned or made a visit when they were really needed  I can get in touch with respiratory team and they’ll check the readings  They will come out if you ask them to… no problem  They’re on the phone and within a couple of days you’ve got all your results  Felt they were able to contact the respiratory team  More appropriate support from health professionals  Able to contact the respiratory team without wasting their time  Valued the ‘connection’ that the telehealth brought with the respiratory nurses  Seen as a benign form of surveillance  I felt I was being monitored, I felt like a connection to the respiratory team  At the other end of the line there was a real person  I know there’s someone at the other end of that line that can help me  Feeling of connection  Providing a connection to their healthcare professionals that they felt comfortable with  Daily sense of connection with the respiratory team, mitigating feelings of loneliness |
|  | Johnston (2) | The nurse wanted to keep an eye on me  Failure to take reading would be noticed by the staff at the call center |
|  | Kim (2) | Ability to quickly connect to a nurse  Receive swift responses to their concerns and questions |
|  | Maric (3) | Felt connected to their healthcare professional  Felt connected to medical care  Notified you if something was wrong |
|  | Piette (2) | Links patients more closely with their care teams  I liked the support that I received using the assessment calling system |
|  | Rahimpour (5) | Would improve access to medical services  Helpful, really hard to ring up a doctor and get them to come and see you  Improving access to healthcare services  Would improve access to healthcare  Improve access to health care services |
|  | Ure (9) | Facilitation of access to professional advice  Ensured a prompt appointment  It would throw up to somebody right away that you need attention  If something was wrong I’d get a phone call from the surgery  Get hold of a doctor if their readings showed I needed a doctor  A single responsive point of access to support was seen as important in ensuring issues were Dealt with promptly  Overcome barriers to arranging a timely appointment  Enabled them to seek professional help.  Improving access to professional care |
|  | Venter (2) | Results were being monitored regularly by the nurse  The nurse would get in contact if worrying trends developed |
|  | Whitten07 (2) | Increases access to care  Increased contact with providers is primary benefit |
|  | Whitten09 (5) | Allowed for immediate health information  Immediate health information via device  If something comes up, it’ll tell you right away  They can get the news quicker  The nurse could see from the signs you were sick and call |
| Happy/ confident in  nurse advice | Clark (5) | CHAT nurses were great Fluid education and other advice helped a lot  Confidence in the advice CHAT nurse offered  Happy with amount of interaction nurse had with family and others  Expert advice came into our home  The nurses were not pushy |
|  | Delaney (8) | Describe their nurses as ‘‘knowledgeable,’’ ‘‘competent,’’ ‘‘caring,’’ and ‘‘outstanding’’  Trust developed in nurse-patient relationship  My nurse was great, and I trusted her  It was good having the same nurse who knew so much about heart failure  Expertise and caring of cardiac nurse  I looked forward to nurse visits, she took the time to make sure I understood my HF  She [nurse] was always so friendly and caring’  Having the nurse go over it with me |
|  | de Lusignan (5) | Nurse able to address what was bothering patient  Nurse cared about patient as a person  I felt I could talk about anything with the nurse  The nurse knew what she was doing  Nurse was sorting our small problems |
|  | Fairbrother 2012 (8) | Patients commented favorably on the approachability of telemonitoring professionals  Get to know their designated telemonitoring professional and form bonds of trust with them  Personalized help, advice and support provided by telemonitoring professionals  Telemonitoring professionals helpful intermediary between themselves and their GP  Valued relationship continuity within telemonitoring provision  Trusted patient-practitioner relationships developed quickly  Greatly appreciated the efforts of telemonitoring professionals to bridge service difficulties  Professionals facilitated contact with GP and organized anticipatory medicine at home |
|  | Kim (4) | Emotional support (2)  Providing detailed explanations, accurate instructions for device usage (2) |
|  | Nguyen (7) | Received support from nurses needed to start/maintain exercise program  Positive experience with program, especially study nurse interactions  Feedback and motivational support on self-management from nurses  Fostered positive relationship between participants and nurses  Nurses showed genuine interest in participants’ well-being  Nurses motivational techniques reinforce confidence in self-management  Positive nurse–patient collaborative increased engagement in exercise |
| As good/better than in person care | Fairbrother 2012;2013a (6) | Relationship between patients and professionals bridged barriers existing in usual care  Telemonitoring professionals liaise with GPs to arrange medical help  I'd say you get better service  Telemonitoring professional says I think you're needing to speak to the doctor  She's just giving me a warning that she's going to get the doctor to phone me  Better attention with telemonitoring |
|  | Gale (3) | I’m not going to know without it  If they take out tomorrow… and I go back to, have to rewire the panic button up again  Am I going to be calling the girls [nurses] out more, am I going to be in hospital more |
|  | Rahimpour (5) | Feel more comfortable at home  Would prefer to take their measurements themselves  Like our doctors but don’t want to see them too often  Very helpful for people that are homebound  Preferred to use system and take measurements on their own |
|  | Whitten 2007 (2) | No strong belief that in-person is superior to telehealth visits  No services necessarily best delivered in person |
|  | | |
| Patient variables | | |
| Convenient | Bedra (2) | Self-testing took very little time  Program would not interfere with usual activities |
|  | Clark (3) | Decreased travelling (reducing travel to healthcare services)  Fantastic system if you live away from town  Diary is very useful and is used for checking back on GP appointments etc |
|  | Finkelstein 2010 (3) | Self-testing took very little time  Self-testing would not interfere with usual activities  System’s convenience |
|  | Finkelstein 2011 (2) | Self-testing process would not interfere with their usual activities  Able to use the system at least few times a week |
|  | LaFramboise (5) | Convenient (3)  Having Health Buddy in home was not time consuming  Anytime of the day option for interaction was convenient |
|  | Maric (2) | The Web site was useful for patient monitoring  I found the website useful |
|  | Rahimpour (11) | More convenient than other methods of health care delivery  Less travelling  Time saved (2)  Fewer medical visits  Really convenient (2)  Don’t have to travel frequently to see doctor, he’s got all records and data  Convenience  Convenient to have HTMS available  Don’t need to visit doctor as often |
|  | Ure (2) | Avoiding the need to travel to the surgery  Prescriptions sent to chemist and delivered direct |
|  | Whitten 2009 (2) | Easier and more convenient  Enjoyed the luxury of staying in their homes |
| Peace of mind | Bedra (2) | Felt safer knowing that they are monitored by the system at home  Feel safer while monitored by the system |
|  | Delaney (2) | Made patients feel secure  I feel safe with the monitor. It takes my weight and blood pressure every morning |
|  | Fairbrother 2012;2013a (6) | Sense of reassurance in having someone ‘watching over them’  It makes you feel like somebody’s looking after you  You’ve got the confidence that they're going to get something done  Provided a sense of reassurance and support  When I’ve got it bad it’s great to know that you can just take a reading  Reassurance of feeling constantly ‘watched over’ by telemonitoring professionals |
|  | Fairbrother 2013b (6) | Felt reassurance  Sort of reassurance that Big Brother’s watching me and even perhaps they can look after me  It’s a safety net  Felt reassurance in having someone watching over them  Virtual safety net that provided peace of mind  Felt reassurance arising |
|  | Gale (16) | Peace of mind (4)  Brought them ‘peace of mind’  It settles your mind more than anything…  It does save me a lot of aggro  You think to yourself, I’m having a good day today  Felt by the participants to be really important for managing anxiety and depression  It’s stopped me pressing the panic button so often  I’ve been more relaxed, if you like, and content  Peace of mind syndrome, it relaxes you a lot  Alleviate feelings of anxiety or panic  Brought feelings of peace of mind  Greater peace of mind (2) |
|  | Radhakrishnan (4) | Sense of security in being monitored by telehealth daily  Felt reassured with daily telehealth monitoring  Sense of security/ peace of mind  Reassurance to family members |
|  | Rahimpour (10) | Improved peace of mind  Could give them peace of mind  Peace of mind (4)  Don’t have to worry about my health as much  Definitely keep a lot of people less worried  By cutting down the stress, it takes their fears away  More relaxed because they know exactly what’s happening to them |
|  | Ure (3) | Reassurance of being monitored  The telemonitoring system ‘watched over’ and ‘looked after’ them  Reassured by the idea that the system could detect impending exacerbations |
|  | Venter (2) | Felt reassured by the presence of the monitor in their home  Increased sense of confidence and well-being |
|  | Whitten 2009 (4) | Worried less in general  Felt safer  Decreased worry  Felt more confident when participating in telehealth monitoring |
|  | | |
| Technology-related | | |
| Ease of use | Antoniades (3) | Found the home monitoring system easy to use  Describing the telemonitoring system as easy to use  Comfortable using home monitoring in their care |
|  | Bedra (2) | Not complicated to use the telecare device  Not difficult to use the keypad |
|  | Clark (2) | Ease of getting connected  Found telecare devices easy to use |
|  | Finkelstein 2010 (2) | Not difficult to work with the computer  System’s ease of use |
|  | Finkelstein 2011 (2) | Controls were simple  Self-testing process was not complicated |
|  | Kulshreshtha (2) | Equipment was easy to use  Equipment was simple and easy to use |
|  | LaFramboise (10) | Technologically easy to use (3)  Found the Health Buddy easy to use  Health Buddy was easy to use  Found the technology relatively easy to use  Health Buddy was not technologically intimidating  Health Buddy did not present technological challenges  Anyone can use health buddy  Positive comments related to technological ease of use |
|  | Lovell (3) | Patients responding favorably on its ease of use  All patients (100%) found the system easy to use  Few or no problems with the operation of the system |
|  | Maric (4) | Web site was easy to use (4) |
|  | Nahm (2) | eHealth program was easy to use  Design of the prototype Web module was evaluated favorably |
|  | Piette (2) | In general, I felt it was easy to use the assessment calling system  Messages were easy to understand |
|  | Rahimpour (8) | Extremely easy to use the system  Found the system easy to use  System is easy to use  The system was easy to use  It looks quite simple and easy  Majority stated they thought that they could use the system  Thinking being able to use the system  Feeling not anxious when thinking about using the system |
